# Subwavelength InSb-based Slot wavguides for THz transport: concept and practical implementations

**DOI:** 10.1038/srep38784

**Published:** 2016-12-07

**Authors:** Youqiao Ma, Jun Zhou, Jaromír Pištora, Mohamed Eldlio, Nghia Nguyen-Huu, Hiroshi Maeda, Qiang Wu, Michael Cada

**Affiliations:** 1 Department of Electrical and Computer Engineering, Dalhousie University, Halifax, NS B3J 2X4, Canada; 2 Institute of Photonics, Faculty of Science, Ningbo University, Ningbo 315211, China; 3 Nanotechnology Centre, VSB Technical University of Ostrava, Ostrava-Poruba 708 33, Czech Republic; 4 Faculty of Information Engineering Department of Information and Communication Engineering, Fukuoka Institute of Technology, Fukuoka 811-0295, Japan; 5 Department of physics and electrical engineering, Northumbria University, Newcastle NE1 8ST, United Kingdom

## Abstract

Seeking better surface plasmon polariton (SPP) waveguides is of critical importance to construct the frequency-agile terahertz (THz) front-end circuits. We propose and investigate here a new class of semiconductor-based slot plasmonic waveguides for subwavelength THz transport. Optimizations of the key geometrical parameters demonstrate its better guiding properties for simultaneous realization of long propagation lengths (up to several millimeters) and ultra-tight mode confinement (~λ^2^/530) in the THz spectral range. The feasibility of the waveguide for compact THz components is also studied to lay the foundations for its practical implementations. Importantly, the waveguide is compatible with the current complementary metal-oxide-semiconductor (CMOS) fabrication technique. We believe the proposed waveguide configuration could offer a potential for developing a CMOS plasmonic platform and can be designed into various components for future integrated THz circuits (ITCs).

Metal nanostructures allow the guiding and manipulation of electromagnetic (EM) field beyond the diffraction limit and are widely considered to be the most promising candidate for the realization of nanoscale optical components and integrated photonic circuits (IPCs)^1^. As the subwavelength information carrier, the metal structures can support the well-known surface plasmon polaritons (SPPs), which are the EM waves coupled to the electron oscillations and propagating along the interface between the dielectric and metallic materials. Such modes have found applications in areas of on-chip waveguiding, biosensing, near-field microscopy and magneto-optic data storage^2^,^3^,^4^,^5^,^6^,^7^. The metal based SPPs devices could provide the nanoscale mode confinement, but high loss is inherent in metal optics and it further increases when the mode sizes are downscaled into the subwavelength level. Basically the mode confinement of SPPs becomes stronger when the operating frequency is closer to the intrinsic plasma frequencies of metals, with more field of SPPs distributed in the vicinity of metal surface thus leading to a larger propagation loss, which suggests a potential leverage to balance the tradeoff between mode confinement and loss by tuning the plasma frequencies of metals^8^. However for noble metals i.e. gold or silver, it is very difficult to alter their plasma frequencies due to rather fixed large carrier concentrations (~10^22^ cm^−3^). On the other hand, plasmonic devices using metals are not compatible with the industry fabrication process, such as complementary metal-oxide-semiconductor (CMOS) technology, which allows the low-cost fabrication of large-scale photonic structures and their integration with nanoscale electronics^9^.

At the same time, an alternative plasmonic material i.e. semiconductors (SCs) offers a more versatile method to engineering the propagation properties of SPPs than metals, in which the free carriers can be controlled by doping, resulting in a plasma frequency typically in the terahertz (THz) domain^10^. Semiconductors have permittivities at the THz range close to those of metals at the optical range. Unlike metals, the plasmonic characteristics of doped SCs can not only be tailored by controlling surface patterns^11^ but also the carrier concentration^12^,^13^ – degrees of freedom that are unavailable in metal materials. Such unique properties have made SCs attractive for various intriguing applications in advanced plasmonic THz systems^14^.

In comparison with visible and infrared (IR) waves, the THz radiation can penetrate into many materials without causing any damage because of its low photon energy. Therefore the THz technology is now receiving an extensive attention with great promises for wide applications such as material characterization^15^, study of electronic coherence of SCs^16^, biochemical sensing^17^ and highly integrated THz circuits (ITCs)^18^. Similar to IPCs, the development of ITCs leads to a new generation of fast, low-loss and on-chip subwavelength THz devices. In this regard, various designs of plasmonic THz structures including waveguides^19^, detectors^20^, sources^21^, resonators^22^ and sensors^23^ have been proposed and demonstrated. For example, the so-called semiconductor-dielectric-semiconductor (SDS) waveguide was intensively studied recently, showing a great potential to build ultra-compact THz plasmonic elements^24^,^25^. However, the SDS waveguide cannot possess a lateral confinement and thus lacks an enriched mode spectrum. To overcome this shortcoming, a THz SC slot waveguide, with a mode area smaller than λ^2^/256, has been recently proposed^26^. This type of a waveguide could provide a stronger mode confinement, but the asymmetric refractive index distribution would induce the mode leakage for a relatively larger slot width, which in turn restricts its degree of freedom for further design. Meanwhile, the THz slot waveguide with stubs was experimentally investigated and explored for detecting biological samples^23^.

Although the guiding properties of SDS and slot waveguides have been studied^24^,^25^,^26^, a more general three-dimensional (3D) SC slot waveguide with fabrication compatibility to the standard CMOS micro-electronic technology has not yet been systematically investigated and reported (the advantages of proposed structure over those reported in refs 24, 25, 26 have been explained in detail and shown in the Supplementary Information). Moreover, the extended discussion about its practical applications has yet to be explored. Therefore in this paper we conduct a comprehensive investigation on a 3D SC slot waveguide with extended applications at THz frequencies. The detailed modal analysis demonstrated that the proposed waveguide offers a superior capacity for low-loss THz transport with propagation lengths reaching up to several millimeters at a subwavelength level and with a mode area of λ^2^/530 at 1 THz. The structure is compatible with the SC fabrication technologies and is expected to be an interesting alternative configuration to realize the ultra-compact devices and sensors for THz applications.

## Results

**Figure 1:**
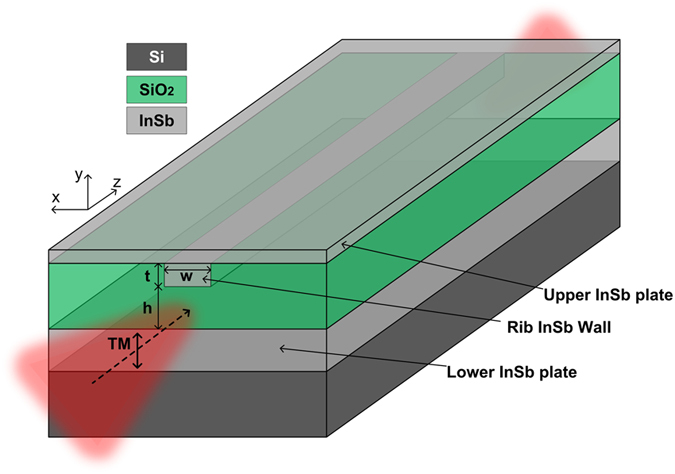
Schematic diagram of 3D view of proposed slot plasmonic THz waveguide.

A schematic diagram of a 3D view of the proposed SC slot waveguide is shown in Fig. 1. All the characteristics of the structure and the coordinate system are depicted in the figure. In our design, the material for the guiding layer is selected as silica (SiO_2_) due to its excellent transparency and relatively low reflectivity at THz frequencies^27^. On the other hand, the semiconductor material is chosen as indium antimonide (InSb) because it has a narrow energy gap and a large electron density, which has been demonstrated to support low-loss tightly-confined THz SPP modes^28^. The frequency/temperature-dependent permittivity of InSb is described by the Drude model^29^:


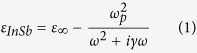


where


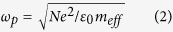


where *ω* is the angular frequency of the incident EM waves, *ε*_∞_ is the high-frequency permittivity, γ is the damping constant, *ω*_*p*_ is the plasma frequency, *e* is the electron charge, *ε*_0_ is the vacuum permittivity, *m*_*eff*_ is effective mass of a free carrier and *N* is the intrinsic carrier density which is temperature-dependent and can be expressed as^30^


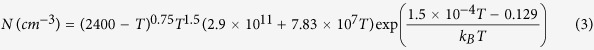


where *T* and *k*_*B*_ are the operating temperature and the Boltzmann constant, respectively. In this paper, SiO_2_ is assumed to be temperature-independent and lossless with a refractive index of 2.1 at THz frequencies of interest^27^. The structure is assumed to be illuminated by a TM-polarized light source.

It should be pointed out that the materials adopted in our design (i.e. Si, SiO_2_ and InSb) and their corresponding synthesis techniques are known to be fully compatible with the standards in the CMOS process^31^. The proposed fabrication process starts with the deposition of InSb and SiO_2_ films on a Si substrate respectively with the ratio frequency magnetron sputtering^32^ and PECVD (plasma enhanced chemical vapor deposition)^33^ techniques. A rectangular groove on the upper SiO_2_ surface is then patterned by using the electron-beam lithography (EBL). Finally the superstrate InSb film is deposited on top of the patterned SiO_2_ layer to form the proposed THz slot waveguide.

**Figure 2:**
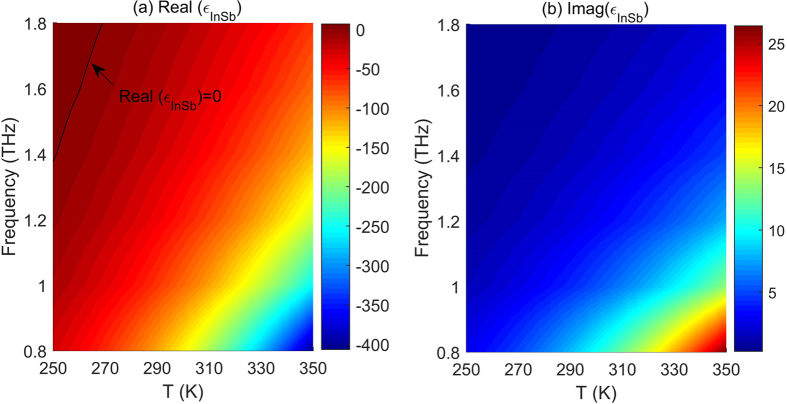
Contour plots of (**a**) real and (**b**) imaginary parts of permittivities of InSb versus both temperature *T* and frequency *f*. Black line shown in (a) is for Real *(ε*_*InSb*_) = 0.

Before examining the modal properties of the proposed structure, the dependence of the permittivity of InSb (*ε*_*InSb*_) on the operating temperature (*T*) and incident frequency (*f*) is plotted in Fig. 2. From Fig. 2(a) it is noted that the real part of *ε*_*InSb*_ i.e. real (*ε*_*InSb*_) is divided into two parts by the black line representing the case of real (*ε*_*InSb*_) = 0, which are real (*ε*_*InSb*_) >0 and real (*ε*_*InSb*_) <0. It is known that the implementation of plasmonic components requires a material with a negative real part of permittivity. InSb can thus act as a plasmonic material when operated at certain temperatures and frequencies. From Fig. 2(a) it is also noted that the tunable range of real (*ε*_*InSb*_) increases with decreasing the frequency and increasing the temperature. For example, when the temperature changes from 250 *K* to 350 *K*, the value of real (*ε*_*InSb*_) varies from −29.9 to −406.9 at a frequency of 0.8 THz, with a tuning range five times larger than that at a frequency of 1.8 THz (for this case the value of real (*ε*_*InSb*_) only varies from 6.6 to −68.1). However, this extended tuning range is achieved at the cost of the relatively larger attenuation loss, as depicted in Fig. 2(b). Therefore, carefully selecting the operating temperature and frequency is crucial to simultaneously achieve a lower loss and a better tunability.

**Figure 3:**
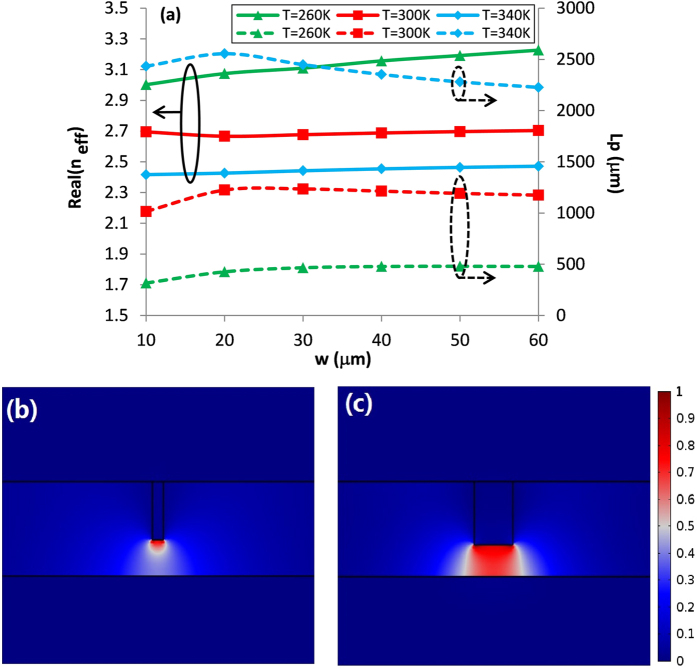
(**a**) Dependences of modal properties of fundamental SPPs mode at frequency of 1 THz for different width *w* and temperature *T*. Normalized electric field abs(*E*_*y*_) profiles for (**b**) *w* = 5 μm and (**c**) *w* = 20 μm at frequency of 1 THz.

We now turn to investigate the dependence of the modal properties on the geometrical parameters (*t, h* and *w*, as shown in Fig. 1). The modal characterization is an essential step to design the waveguide devices to obtain the optimized structural parameters. Figure 3(a) shows the mode effective index and the propagation length of the fundamental SPP mode for different temperatures *T* when the value of *w* varies from 10 to 60 μm, while the frequency is fixed at *f* = 1 THz (the wavelength is 300 μm). In this simulation, the other parameters are selected as follows: *h = *15 μm and *t* = 30 μm. From Fig. 3(a) it is found that the value of real (*n*_*eff*_) increases with the increase of *w*, which is physically reasonable due to the larger overlap between the mode field and the material of SiO_2_. While the propagation length is shown to increase at first then decreases exhibiting maximum values at certain values of *w*, i.e. around 20 μm, where the propagation length (*L*_*p*_ = 1.24 mm) is at least 2 times longer than that reported in ref. 26 with *L*_*p*_ = 0.3 mm. This phenomenon can be understood as follows: For a larger value of *w (w* > 20 μm), a larger portion of power will be in contact with the rib and lower InSb walls resulting in a larger dissipation loss. Thus the propagation length will accordingly become shorter. On the other hand, if the value of *w* is smaller than 20 μm, the electric field (*E*_*y*_) will be strongly localized around the end-face of the rib InSb wall, as depicted in Fig. 3(b), forming a localized-SPP-like (LSPP-like) mode with a relatively higher loss^34^. While for a moderate value of *w (w* = 20 μm), as shown in Fig. 3(c), the electric filed is shown to be highly confined inside the TPX subwavelength slot region. Therefore, in the following, the value of *w* is set to be 20 μm to achieve a relatively long propagation length.

**Figure 4:**
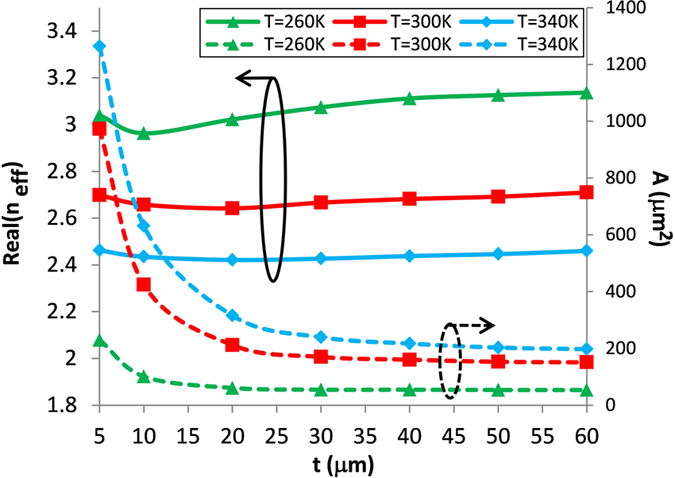
Modal properties of fundamental SPP mode as function of parameter *t* with different temperature *T*.

Figure 4(a) shows the influence of the parameter *t* on the mode effective index and the mode area with the temperature *T* at a frequency of *f* = 1 THz. In this simulation, the waveguide parameters are chosen as *h = *15 μm and *w* = 20 μm. A smaller mode area corresponds to a stronger mode confinement. The waveguide acts as a parallel-plate waveguide (PPWG)-like when *t* is very small, with the field concentrated between the two parallel InSb (lower and upper) walls, resulting in a larger mode area as well as a mode effective index. As *t* increases, the mode confinement will be improved in the lateral direction due to the fact that the effective index of the slot (between the rib and lower InSb walls) waveguide mode is larger than that of the PPWG mode. Thus the field will be tightly constrained into the slot region, as shown in Fig. 4, giving rise to a stronger mode confinement (i.e. smaller mode area) and a larger mode effective index. From Fig. 4 one can also see that the mode area is not sensitive to *t* when *t* is larger than 30 μm. Therefore we will select *t* as 30 μm in the following discussion to maintain a compact physical size. The mode area for case of *t* = 30 μm at room temperature is 170 μm^2^ equaling to λ^2^/530, which is 130 times smaller than that of the diffraction limited mode area, i.e. λ^2^/4.

**Figure 5:**
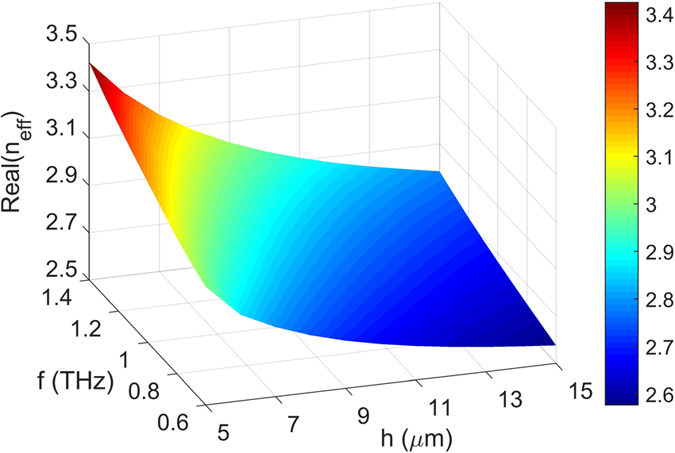
Real parts of effective indices of fundamental SPP mode as functions of frequency *f* and parameter *h.*

The modal characteristics are also dependent on the parameter *h* and the incident frequency *f*. Figure 5 depicts the simulation results for the mode effective index versus *f* and *h*. The parameters used in this simulation are *T* = 300 K, *w* = 20 μm and *t* = 30 μm. It is clear that the value of real (*n*_*eff*_) increases with increasing *f* for a given *h*, which is consistent with the permittivity variation shown in Fig. 2(a). Moreover, one can see that the value of real (*n*_*eff*_) increases with the decrease of *h* for a fixed *f*. A smaller value of *h* results in a stronger mode confinement, thus leading to a larger mode effective index. For example, at a frequency of 1 THz, the mode effective indices of the waveguide with *h* = 15 μm and *h* = 5 μm equal to real (*n*_*eff*_) = 2.667 and real (*n*_*eff*_) = 3.182, respectively.

The results discussed above indicate that the proposed SC slot waveguide with optimized geometrical parameters could guide the THz wave in a subwavelength region (hundreds times smaller than the diffraction-limited mode area) with a long propagation length (several millimeters), making it potentially a basic structure for the design of novel types of ITCs devices.

### Discussion about InSb-based slot plasmonic waveguides for practical applications

**Figure 6:**
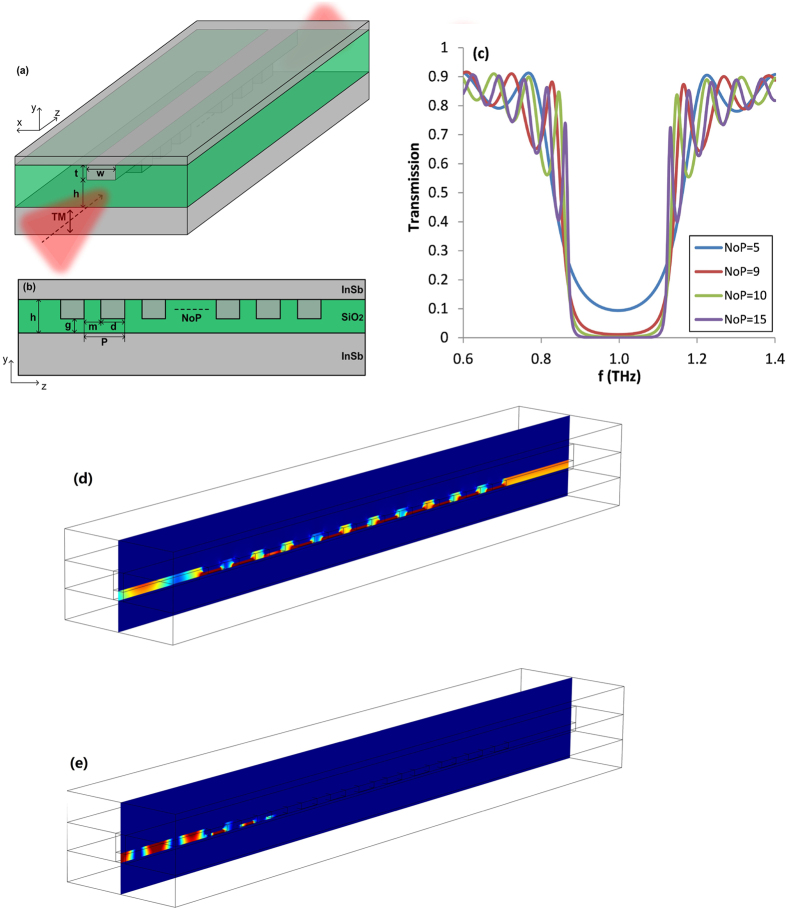
Schematic diagrams of (**a**) 3D view and (**b**) side view in y-z plane of Bragg reflector based on proposed InSb slot plasmonic waveguide. (**c**) Transmission spectra of Bragg reflector consisting of 5, 9, 10 and 15 periods. Contour profiles of normalized electric field abs (*E*_*y*_) patterns at frequencies of (**d**) 0.6 THz and (**e**) 1 THz.

Owning to the superior guiding performance of the InSb slot plasmonic waveguide, below we focus on investigating its practical implementations and applications. As illustrated in Fig. 5, the waveguide with different parameter h leads to the contrast of mode effective indices. Obviously such contrast can be realized, as shown in Fig. 6(a,b), by periodically selecting different parameters *h* and *g (h* > *g*) along the propagating direction of surface plasmon wave (SPW). This periodical index modulation in turn forms the well-known photonic band gap (PBG)^35^, which is commonly utilized as filters for achieving wavelength selective functions. A variety of SPP filters have been intensively studied in the optical region^36^,^37^,^38^ but for THz frequencies they are rarely investigated. Additionally, these SPP filters are passive, indicating the filtering characteristics depend on the structural parameters, thus their filtering functions will be unchangeable once the devices are fabricated.

As an example of the design, the central Bragg frequency is set to be *f*_*b*_ = 1 THz. In this simulation, other parameters are chosen as *T* = 300 K, *w* = 20 μm, *t* = 30 μm, *h* = 15 μm and *g* = 5 μm. According to the Bragg condition, i.e. 2 *m* Real (*n*_*eff,h*_) + 2*d* Real (*n*_*eff,g*_) = *λ*_*b*_, where Real (*n*_*eff,h*_) = 2.667 and Real (*n*_*eff,g*_) = 3.182, it is known that the Bragg scattering takes place around *λ*_*b*_ by selecting parameters of *m* = 20 μm and *d* = 30 μm (the grating period is denoted as *P* = *m* + *d* = 50 μm). By adopting these designed parameters, Fig. 6(c) shows the corresponding transmission spectra of the structure. As expected one can see that there is a PBG around *f* = 1 THz when a finite number of period (NoP) is considered. In principle, the Bragg scattering exists for any NoP; for the case of the proposed structure, it is found that the minimum NoP required to obtain the transmission less than 1% is 10. However, the increase in NoP gives rise to a higher propagation loss, as shown in Fig. 6(c). In addition, one can also see that some sidelobes appear on both sides of the PBG, which may be due to the light scattering at the abruptly disappearing boundary on both ends of the Bragg periodicity. To further verify the above results, the normalized electric field (abs (*E*_*y*_)) patterns of the structure at frequencies of 0.6 THz and 1 THz were investigated and are summarized in Fig. 6(d) and (e), respectively. From Fig. 6(d) one can see that if the incident frequency is not within the band gap, the SPW is mainly guided through the structure and weakly affected by the presence of the Bragg grating. However, when the incidence frequency is located in the band gap, the transmission of SPW through the structure is forbidden, as depicted in Fig. 6(e). The results illustrate that the proposed structure can act as a stop-band filter.

**Figure 7:**
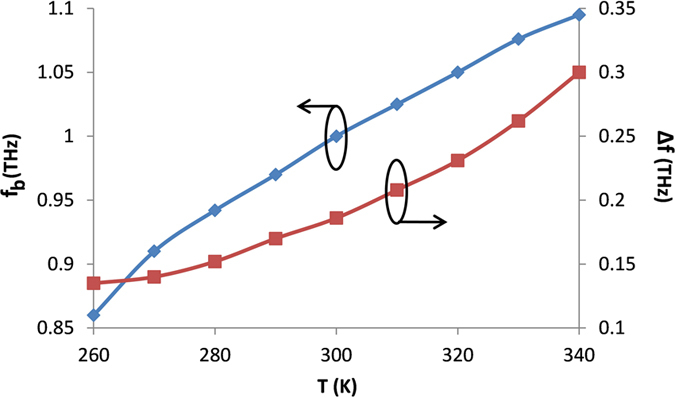
Influence of temperature *T* on central Bragg frequency (*f*_*b*_) and bang gap (∆*f*).

The transmission of the Bragg reflector is also highly dependent on the operating temperature. It is important to investigate this because such a dependency offers a possibility of enabling thermo- or electro-plasmonic modulation of a device. Figure 7 shows the effect of the temperature on the central Bragg frequency (*f*_*b*_) and the band gap (*∆f*). The parameters used are *w* = 20 μm, *t* = 30 μm, *h* = 15 μm, *g* = 5 μm, NoP = 10, *m* = 35 μ*m* and *d* = 40 μ*m*. It is clear that both *f*_*b*_ and *∆f* increase with increasing *T*. For example, *f*_*b*_ (*∆f*) varies from 0.86 THz (0.135 THz) to 1.095 THz (0.301 THz) for *T* changing from 260 K to 340 K. The influence of temperature on the characteristics of FBG is mainly attributed to the variation of the mode effective index, resulting from the change of the permittivity of InSb.

**Figure 8:**
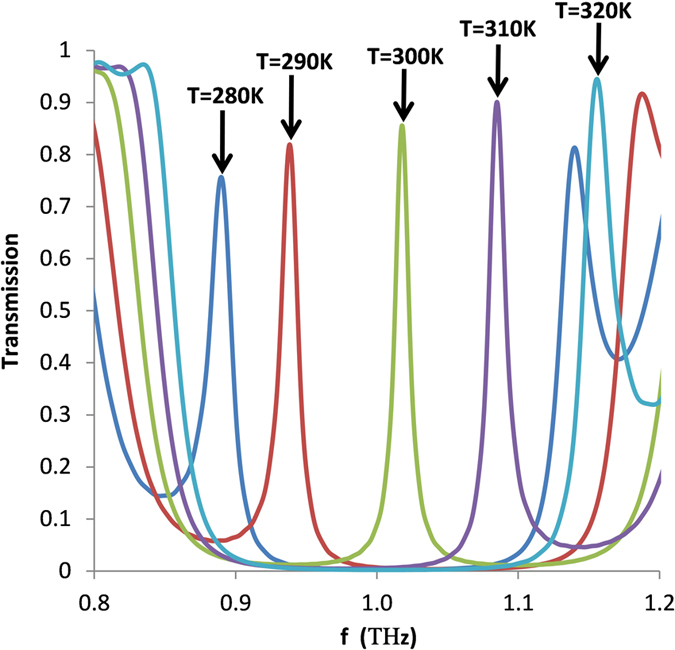
Transmission spectra of the Bragg reflector structure with FP cavity in middle of Bragg grating for different temperature *T*.

The periodic index perturbation forms a wavelength specific dielectric mirror, so a Fabry-Perot (FP) cavity can be created by introducing a phase shift in the middle of the Bragg grating. The phase shift leads to a sharp resonance peak within the band gap of the transmission, suggesting the Bragg reflector with FP cavities is preferred for designing temperature sensors compared to the Bragg reflector structures. Figure 8 shows the transmission spectra with different *T* when the 6 th period is designed as *h* = *g = *15 μm. In this simulation other parameters are *w* = 20 μm, *t* = 30 μm, *h* = 15 μm, *g* = 5 μm, NoP = 11, *m* = 35 *μm* and *d* = 40 *μm*. As can be seen, the peak frequency becomes higher as *T* is increased, illustrating a temperature sensitivity of 6.6 × 10^−3^ THz/K, which is much higher than that of 1.425 × 10^−3^ THz/K reported in ref. 24 and comparable to that of 7.5 × 10^−3^ THz/K achieved in ref. 39. The shift of the peak frequency is caused by the variation of real (*ε*_*InSb*_) induced by the temperature. In addition, one can see that the peak frequency will move out of the band gap if one chooses a higher or a lower temperature. However, this limitation can be addressed by increasing the difference between Real (*n*_*eff,h*_) and Real (*n*_*eff,g*_), or in other words, by increasing and decreasing the values of *h* and *g*, respectively.

Another attractive application of the Bragg reflector with a FP cavity is to develop integrated biosensors since it offers a large overlap between the cavity mode and the analyte, assuming that the analyte liquid is transported into the microcavity through an integrated microfluidic^40^. Moreover, it exhibits a narrow peak indicating a high measurement precision. However, a scheme for biosensing is desired to eliminate the cross-sensitivity between the refractive index and temperature variations^41^.

**Figure 9:**
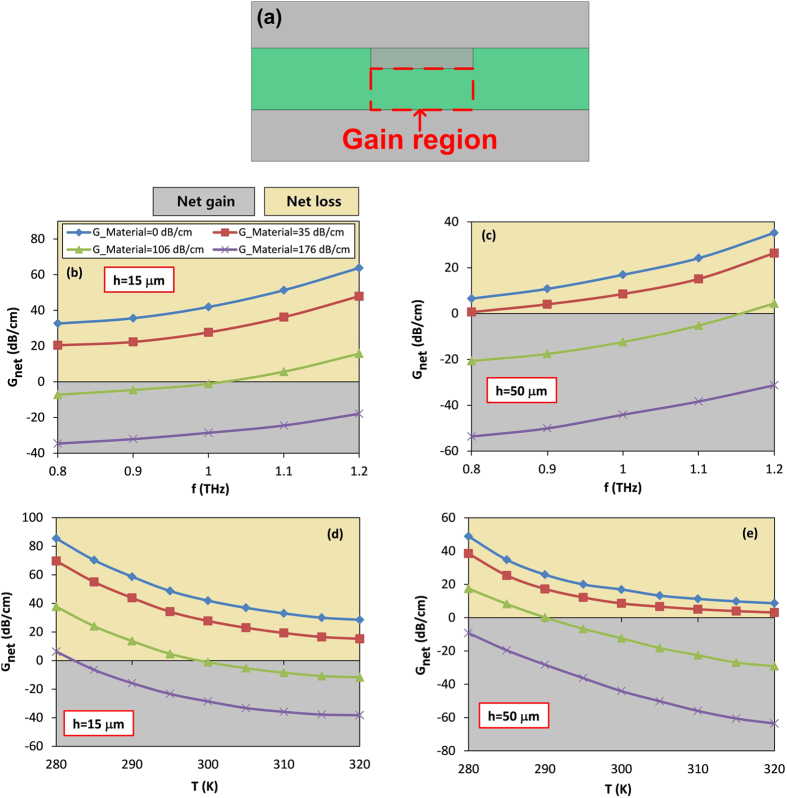
(**a**) Cross-section view of proposed slot plasmonic THz waveguide with gain medium introduced in slot region (marked by red dashed line). Net gain (or loss) versus frequency for different material gain for case of (**b**) *h* = 15 μm and (**c**) *h* = 50 μm. Net gain (or loss) versus temperature for different material gain for case of (**d**) *h* = 15 μm and (**e**) *h* = 50 μm.

Even though the proposed SC slot waveguide provides a way to achieve the low-loss THz transport, the intrinsic loss still limits its applications for the long-distance THz interconnect. One of the most effective approaches to address this limitation is to introduce a medium with gain, such as the boron-doped silicon^42^ or neodymium-doped silica^43^ or graphene^44^. The gain medium can be introduced inside the slot region, as shown in Fig. 9(a), and the pump can be fed in either optical or electrical ways^45^,^46^. In our simulation the reported material gain values are assumed, e.g. *G*_*material*_ = 35 dB/cm, 106 dB/cm and 176 dB/cm^47^. Figure 9(b) and (c) show the evaluation of the net gain (or loss) versus the frequency with *h* = 15 μm and 50 μm, respectively. The other parameters are set as *t* = 30 μm, *w* = 20 μm and *T* = 300 K. As expected from Fig. 9(b), it is found that the loss of the SC slot waveguide becomes smaller as *G*_*material*_ increases, showing that it is possible to compensate the intrinsic loss of the SPP mode. When the value of *G*_*material*_ is large enough (i.e. *G*_*material*_ = 176 dB/cm), the value of *G*_*net*_ becomes negative (as explained by eq. (6) in section of Methods), or in other words, a pure gain is generated. Another point should be noted is that the influence of the gain medium on the propagation properties is temperature dependent. Figure 9(d) and (e) show the calculated net gain (or loss) with *h* = 15 μm and 50 μm, respectively, as a function of temperature *T*, by using parameters of *t* = 30 μm, *w* = 20 μm and *f* = 1 THz. Similarly, the pure loss converts to the pure gain by enhancing the material gain, and the lossless propagation can be realized at certain threshold temperature (*T*_*ts*_, where the value of *G*_*net*_ = 0). If the temperature is higher than *T*_*ts*_, a pure gain is produced. In addition, as shown in Fig. 9(d), the value of *T*_*ts*_ decreases with the increase of the material gain. For instance, when *G*_*material*_ = 106 dB/cm and *G*_*material*_ = 176 dB/cm, the threshold temperatures are 299 K and 283 K, respectively. Furthermore, from Fig. 9(b–e) it is seen that one can obtain a much higher pure gain by choosing a larger slot height. For example, with the same material gain of *G*_*material*_ = 106 dB/cm, the pure gain for device with *h* = 50 μm (*G*_*net*_ = −12.4 dB/cm) is at least ten times enhanced compared to that with *h* = 15 μm (*G*_*net*_ = −1.14 dB/cm) at *T* = 300 K and *f* = 1 THz. However, it should be noted that such increased pure gain is obtained by scarifying the mode confinement. The selection of structural parameters should be subject to the tradeoff between the pure gain obtained and mode confinement.

## Conclusion

In conclusion, we have theoretically demonstrated low-loss InSb-based plasmonic slot waveguides for THz transport, which is compatible with the current CMOS process and could simultaneously provide a subwavelength mode confinement and a long propagation length. We have shown that the mode propagation length can reach several millimeters at a mode area below λ^2^/530. Studies on Bragg reflectors and microcavity sensors further reveal potential capabilities of the proposed waveguide configuration in developing components for ITCs. Because of promising guiding properties, the proposed waveguide possesses a great potential for the implementation of THz components integrated on a chip, thus opening attractive venues in various applications.

## Methods

In the manuscript, the numerical calculations of the modal and propagation properties of the structure were performed by means of the finite element method (FEM) software package (COMSOL Multiphysics) with the Radio Frequency (RF) module. The well-known perfectly matched layers (PMLs) were employed at the calculation domain boundaries to absorb the reflection of the outgoing electromagnetic waves. Convergence tests are done to assure the meshing and boundaries do not affect the solutions. The modal properties of the structure is characterized by a complex propagation constant *β = β*_*r*_ + *β*_*i*_. Here, *β*_*r*_ and *β*_*i*_ are the phase and attenuation constants, respectively. Then the real part of the effective mode index is calculated by Real (*n*_*eff*_) = *β*_*r*_/*k*_*0*_, where *k*_*0*_ is the vacuum propagation constant, and the propagation length is calculated as *L*_*P*_ = 1/2*β*_*i*_ = *λ*/[4*π*Im(*n*_*eff*_)]. The key parameter demonstrating the mode confinement capability is the mode area A, which is defined as the ratio of the total mode energy density *W*_*t*_ and the peak energy density *W*_*p*_ (x, y), which can be expressed by the formulas^2^:


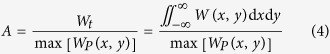



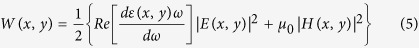


where |E(x, y)|^2^ and |H(x, y)|^2^ respectively denote the intensity of electric and magnetic fields, *ε(x, y)* is the electric permittivity and *μ*_*0*_ is the vacuum magnetic permeability.

The net mode gain (or loss) is determined by two factors, the mode propagation loss and the mode fraction confined within the gain material, and can be evaluated by^48^


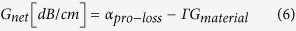


where *G*_*net*_ is the net mode gain (or loss), *α*_*pro*−*loss*_ = 2 × *β*_*i*_ × 4.34 is the mode propagation loss of the structure without introducing the gain medium (the factor of 4.34 converts the value of loss from 1 /cm to dB/cm), *Γ* is the fraction of the THz power overlapping with the gain medium, and *G*_*material*_ is the gain of the bulk material. Note that the net gain and loss occur while *G*_*net*_ < 0 (i.e. *ΓG*_*material*_ > *α*_*pro*−*loss*_) and *G*_*net*_ > 0 (i.e. *α*_*pro*−*loss*_ > *ΓG*_*material*_), respectively.

## Additional Information

**How to cite this article**: Ma, Y. *et al*. Subwavelength InSb-based Slot wavguides for THz transport: concept and practical implementations. *Sci. Rep.* **6**, 38784; doi: 10.1038/srep38784 (2016).

**Publisher's note:** Springer Nature remains neutral with regard to jurisdictional claims in published maps and institutional affiliations.

## Supplementary Material

Supplementary Information
