# Supplementary material for: Subwavelength InSb-based Slot wavguides for THz transport: concept and practical implementations
Source: Sci Rep. 2016 Dec 7;6:38784. doi: 10.1038/srep38784 (PMC5141467; doi:10.1038/srep38784)
Supplement: Supplementary Information [file srep38784-s1.pdf]

Supplementary Information for:

Subwavelength InSb-based Slot wavguides for THz transport: concept and practical implementations

Youqiao Ma[1,*], Jun Zhou[2], Jaromír Pištora[3], Mohamed Eldlio[1], Nghia Nguyen-Huu[1], Hiroshi Maeda[4], Qiang Wu[5] and Michael Cada[1]

[1]Department of Electrical and Computer Engineering, Dalhousie University, Halifax, NS B3J 2X4, Canada

[2]Institute of Photonics, Faculty of Science, Ningbo University, Ningbo 315211, China

[3]Nanotechnology Centre, VSB Technical University of Ostrava, Ostrava-Poruba 708 33, Czech Republic

[4]Faculty of Information Engineering Department of Information and Communication Engineering, Fukuoka Institute of Technology, Fukuoka 811-0295, Japan

[5]Department of physics and electrical engineering, Northumbria University, Newcastle NE1 8ST, United Kingdom

*Corresponding Author. Email: mayouqiao188@hotmail.com

S1. Comparison between present work and structures studied in Refs. 24-25

The semiconductor-insulator-semiconductor waveguide (SCISCW) structures discussed in Refs. 24-25 are assumed that there is no spatial variation along x-direction, thus they lack the capacity of lateral mode confinement. In order to make a reasonable comparison with the proposed three-dimensional (3D) SC slot waveguide (SCSW), the counterpart 3D structure for SCISCW in Refs. 24-25 is considered, i.e. the length in x-direction (L) is limited. For example, Suppl. Fig.1 (a-b) shows the normalized electric field (abs(E_y)) distributions for the SCSW and SCISCW modes in an air environment at 1 THz, with the structural parameters of [$h = 15$ μm, $t = 30$ μm, $w = 20$ μm, $L = 200$ μm] and [$h = 15$ μm, $t = 0$ μm, $L = 200$ μm], respectively. It is found that the SCSW is a better alternative to enhance the mode confinement along x-direction, which offers benefits for realizing tight field localization. Suppl. Fig.1 (c) depicts their normalized plots of abs (E_y) along x-axis. The lateral mode width (MW, which is defined as the full width where abs (E_y) decays to $1/e$ of its peak value) of the SCSW mode is approximately three times smaller than that of the SCISCW mode i.e., $MW_{SCSW} = 50$ μm and $MW_{SCISCW} = 160$ μm. However, one should note that such difference of lateral mode confinement can still get more obvious as L increases.

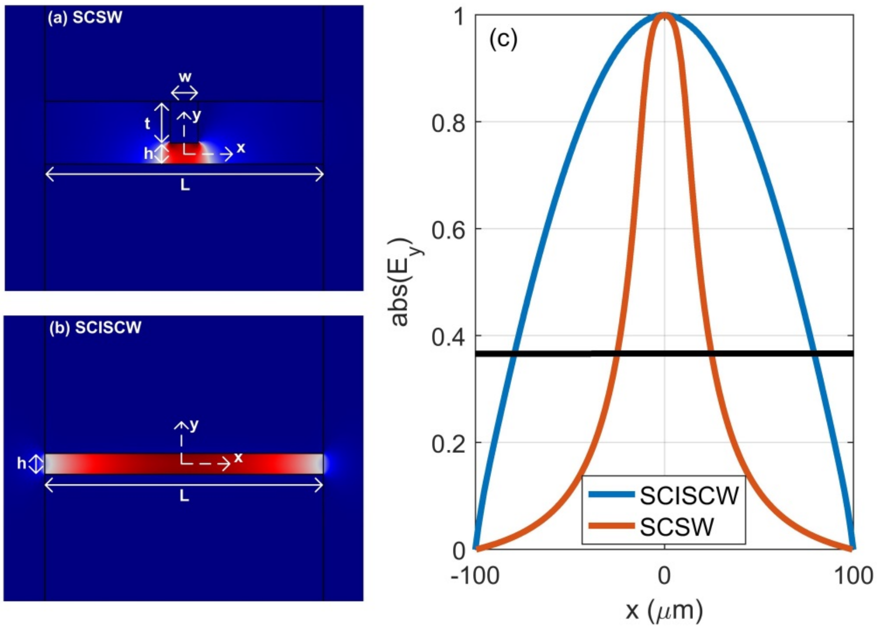

Supplementary Figure 1: Normalized electric field abs (E_y) distributions for (a) SCSW and (b) SCISCW modes at 1 THz with air surrounded. (c) Normalized electric field abs (E_y) plots along x-direction for SCSW and SCISCW modes at 1 THz with air surrounded. Black line represents location where abs (Ey) decays to 1/e of its peak value.

S2. Distinct merit of present work over waveguide reported in Ref. 26

The slot waveguide proposed in Ref. 26 could achieve a subwavelength field localization, however, the dimension of the slot region is strictly limited to prevent the mode substrate leakage. Both the width and height of the slot are shown to be smaller than 7.5 μm for the design in Ref. 26. Such limited slot sizes in turn reduce the power coupling from the conventional dielectric waveguides when they are integrated on the same THz circuits. To explain this limitation, a waveguide coupling scheme is proposed and schematically shown in Suppl. Fig.2 (a), where the power transfers from a Si ridge waveguide (SRW, with a width and a height of D and H, respectively) to the proposed SCSW (with different slot height h).

Before studying the power coupling, the parameters of the SRW is designed. Suppl. Fig.2 (b) and Suppl. Fig.2 (c) respectively shows the effective refractive indices for SRW as functions of D ($H = 80$ μm) and H ($D = 50$ μm) at 1 THz. As shown in Suppl. Fig.2 (b), the fundamental TM mode (TM0) exists for all values of D considered, while for the fundamental TE mode (TE0), it cannot be supported while D is smaller than 59 μm. As known that the SPP mode is TM-polarized, thus the value of D is selected as 50 μm to support only TM mode. In addition, the value of H is fixed to be 80 μm for the fundamental mode operation, as depicted in Suppl. Fig.2 (c). On the other hand, for the SCSW, the parameters of w and t are chosen as the optimized values of 20 μm and 30 μm, respectively.

The power coupling efficiency is determined by the overlap integral of electromagnetic fields between the SRW and SCSW modes. Suppl. Fig.2 (d) illustrates their overlap integral as a function of h at 1 THz. The overlap integral (OI) is defined as [Suppl.1]:

$$\mathrm{OI} = \frac{|\iint E_{SRW}(x,y) \times H^*_{SCSW}(x,y)dxdy|^2}{|\iint E_{SRW}(x,y) \times H^*_{SRW}(x,y)dxdy| \cdot |\iint E_{SCSW}(x,y) \times H^*_{SCSW}(x,y)dxdy|}$$

where E_{SRW} and E_{SCSW} are the electric field components of the SRW and SCSW modes, respectively. As shown in Suppl. Fig.2 (d), the coupling efficiency highly depends on the size of the slot. With the increase of the slot height h, the OI is shown to increase at first and then decreases exhibiting a maximum value at a certain slot height (i.e., around $h = 50$ μm), indicating that the flexible slot sizes are crucial to maximize the power coupling efficiency. Such slot-size dependent coupling, on the other hand, further shows the critical limitation of the waveguide proposed in Ref. 26.

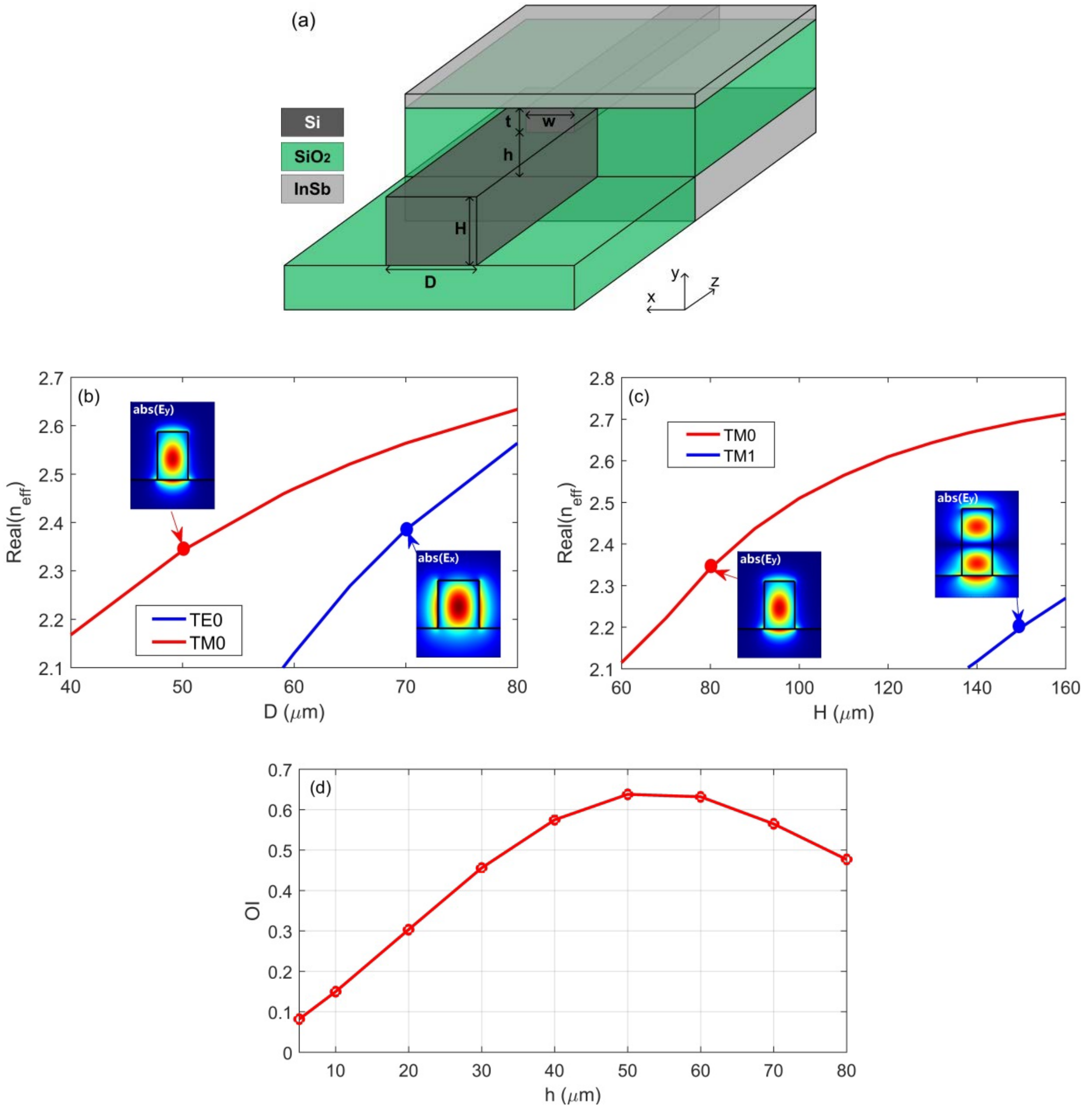

Supplementary Figure 2: (a) Sketch of proposed waveguide coupling scheme from Si ridge waveguide to SC slot waveguide. Effective refractive indices versus (b) width *D* and (c) height *H* of Si ridge waveguide at frequency of 1 THz. Insets in (b-c): normlaized electric field distributions. (d) Overlap integral of electromagnetic fields between SRW and SCSW modes.

Supplementary Reference

[Suppl. 1] Horth, A., Cheben, P., Schmid, J. H., Kashyap, R. & Quitoriano, N. J. Ideal, constant-loss nanophotonic mode converter using a Lagrangian approach. *Opt. Express* **24**,6680-6688 (2016).
